# Draft Genome Sequence of Vibrio parahaemolyticus PSU5579, Isolated during an Outbreak of Acute Hepatopancreatic Necrosis Disease in Thailand

**DOI:** 10.1128/mra.00873-22

**Published:** 2023-01-19

**Authors:** Jason P. LaPorte, Edward J. Spinard, Damian Cavanagh, Marta Gomez-Chiarri, David C. Rowley, John J. Mekalanos, Pimonsri Mittraparp-arthorn, David R. Nelson

**Affiliations:** a Department of Cell and Molecular Biology, University of Rhode Island, Kingston, Rhode Island, USA; b Department of Fisheries, Animal and Veterinary Sciences, University of Rhode Island, Kingston, Rhode Island, USA; c Department of Biomedical and Pharmaceutical Sciences, University of Rhode Island, Kingston, Rhode Island, USA; d Department of Microbiology and Immunobiology, Harvard Medical School, Boston, Massachusetts, USA; e Division of Biological Science, Faculty of Science, Prince of Songkla University, Hat Yai, Thailand; Montana State University

## Abstract

Here, we announce the draft genome sequence of Vibrio parahaemolyticus strain PSU5579, isolated from a shrimp hatchery in southern Thailand during an outbreak of acute hepatopancreatic necrosis disease (AHPND). The genome contains 44 contigs with a sequence length of 5,229,426 bp, 4,861 coding sequences, and a G+C content of 45.3%.

## ANNOUNCEMENT

Acute hepatopancreatic necrosis disease (AHPND), responsible for mass mortality events in shrimp aquaculture systems worldwide, has caused production losses estimated at over $44 billion (1, 2). The etiologic agent of AHPND has been identified as Vibrio parahaemolyticus strains possessing the plasmid pVPA3-1, which encodes the toxins PirA^Vp^ and PirB^Vp^ (3–5). Experiments have demonstrated that V. parahaemolyticus PSU5579 induces >60% mortality in postlarval whiteleg shrimp (Litopenaeus vannamei) when administered via bath immersion (6). The V. parahaemolyticus PSU5579 genome has been sequenced, assembled, and annotated as reported here.

V. parahaemolyticus PSU5579 was originally isolated from an AHPND-afflicted shrimp farm located in southern Thailand (7, 8). A colony of PSU5579, struck from a cryostock maintained in the Nelson laboratory, was inoculated in Luria-Bertani broth supplemented with 2% NaCl (LB20) and grown overnight at 27°C. Genomic DNA, isolated using a Wizard genomic DNA purification kit (Promega), was resuspended in 2 mM Tris-HCl buffer (Bio Basic), quantified on a NanoDrop 1000 spectrophotometer (ND-1000), and checked for quality on a 1% agarose gel stained with ethidium bromide. Total genomic DNA was sheared via sonication (Covaris S220 sonicator), and libraries were prepared using a SMARTer PrepX DNA library kit on a SMARTer Apollo system (TaKaRa Bio, USA).

Paired-end sequencing (2 × 250-bp) was performed at the Rhode Island Genomics and Sequencing Center on an Illumina MiSeq instrument. Sequence trimming was performed using CLC Genomics Workbench version 9.5.3, resulting in 2,743,364 paired-end reads. Assembly was performed using the SPAdes version 3.1.1 genome assembler (9). Contigs with a coverage depth of ≥34 reads were processed using the CLC Microbial Genome Finishing module, with V. parahaemolyticus RIMD 2210633 as a reference. The draft genome, including the complete 69,150-bp plasmid pVPA3-1, consists of 44 contigs (*N*_50_ contig length, 286,064 bp; average contig length, 118,851 bp; longest contig, 532,770 bp; shortest contig, 1,007 bp), with a total sequence length of 5,229,426 bp and a G+C content of 45.3%. Gene annotation with Rapid Annotations using Subsystems Technology (RAST) resulted in 4,861 coding sequences (10–12). Default parameters were used for all software unless otherwise noted.

The genome of V. parahaemolyticus PSU5579 encodes a variety of lytic enzymes, including phospholipases A and C, one extracellular lipase, one chitinase, and nine hemolysins. Several iron acquisition systems were annotated, along with secretion systems that include a type 1 secretion system (T1SS), T2SS, T3SS, T4SS, and two T6SS (13).

Notably, a region of contig 38 (positions 5259 to 537; see “Data availability”) encodes the recently described T6SS VgrG1b module, which consists of the polymorphic nuclease effector (PoNe) and DUF1911 immunity protein (PoNi), as well as an auxiliary VgrG (VgrG1b) (14). The PoNe, PoNi, and VgrG1b proteins encoded by PSU5579 are identical to those first reported in V. parahaemolyticus 12-297/B (GenBank accession no. MYFG00000000).

The genome also encodes a capsular polysaccharide, an RTX toxin, and a beta-lactamase. The circular plasmid pVPA3-1 harbors *pirA-* and *pirB*-like genes on a 3.5-kbp fragment, flanked by transposases. The genome lacks the *tdh* or *trh* genes associated with pathogenicity in humans (15).

### Data availability.

This whole-genome shotgun project has been deposited in DDBJ/ENA/GenBank under accession no. PEBT00000000. The version described in this paper is the first version, PEBT00000000.1. The raw reads were deposited in the Sequence Read Archive (SRA) under accession no. SRX17408971, with BioProject accession no. PRJNA415212 and BioSample accession no. SAMN07818924. The sequence for contig 38 is available under GenBank accession no. NZ_PEBT01000038. The PoNe, PoNi, and VgrG1b proteins are available under protein accession no. WP_029857615, WP_024703575, and WP_236576042, respectively. The plasmid pVPA3-1 sequence was deposited under GenBank accession no. NZ_CM009237.
